# Sigma metrics of Alinity ci system – a study on thirty-nine clinical chemistry and immunoassay parameters

**DOI:** 10.1515/almed-2021-0001

**Published:** 2021-04-01

**Authors:** Fatima Zehra Kanani, Adnan Haider Kazmi, Bushra Kaleem

**Affiliations:** Department of Pathology, Section of Chemical Pathology, The Indus Hospital, Karachi, Pakistan; Indus Hospital Research Centre, The Indus Hospital, Karachi, Pakistan

**Keywords:** accuracy, performance specifications, precision, six sigma, validation

## Abstract

**Objectives:**

Sigma metrics in an invaluable and inexpensive tool used in laboratories to monitor analytical quality of the assays. Alinity ci platform is a relatively recent analytical system launched by Abbott Diagnostics, and as such performance studies on it are few. We have calculated sigma metrics of 39 clinical chemistry and immunoassay analytes on two Alinity ci systems.

**Methods:**

Sigma metrics were calculated using results of method validation studies. Coefficient of variation (CV) was calculated according to CLSI EP 15 guidelines. Bias was calculated using three different methods i.e., proficiency testing material, alternate method comparison with existent analyzers and linearity experiment. Total allowable error limits were kept similar to or less than the ones used in reference studies.

**Results:**

All analytes except blood urea nitrogen (BUN) demonstrated greater than six sigma value across one or more levels and methods. No analyte amongst clinical chemistry and immunoassays was at below three sigma class. Amongst electrolytes, sodium was below three sigma class at two levels by proficiency testing method, although it was above four sigma class by other two methods. Sigma levels obtained were comparable to those reported in previously published studies.

**Conclusions:**

Acceptable sigma metrics were achieved for all clinical chemistry, immunoassays and electrolytes on Alinity ci. Sigma metrics is an objective and well established cost effective tool to tailor internal quality control practices. This study determines sigma metrics for a wide range of high throughput assays. Long term assay performance needs to be monitored.

## Introduction

Sigma metric analysis is a well-established tool for quality assessment in industry which is now being increasingly used in clinical laboratories [1]. It is based on the concept of defects per million opportunities and aims to minimize errors in processes [2]. In the clinical laboratory, the internal and external quality control statistics are often used to calculate measurable values for sigma calculations based on the precision and accuracy of the assay. Clinical chemists and laboratorians have used various approaches to assess and monitor their laboratory performance based on this technique. The precision is generally calculated by the coefficient of variation generated by the daily analysis of the different internal quality control levels used for the assays. The accuracy is studied by calculating the bias, usually from the peer group values on Proficiency Testing schemes, although other approaches have also been used [3], [4]. Another variable in the calculation of Sigma-metrics is the total allowable error (TEα) limit chosen. There are different sources available which have proposed various TEα for analytes based on optimum analytical performance [5], [6], [7], [8], clinical guidelines [9] or biological variation [10]. A summary of some of these available sources is given in Supplementary Table S1.

The major advantage of sigma-metrics is that it provides a quantifiable and measurable tool against which assay performance can be objectively assessed and monitored. Six sigma and above are taken as world class standard, in which there are just under four defects per million opportunities. The minimum acceptable standard for a clinical laboratory assay is a three-sigma level. This approximates to 67,000 defects per million opportunities, below which the assays are not deemed clinically safe for patient testing [11], [12], [13].

Another important benefit of calculating the sigma metrics of the laboratory assays is that as we objectively measure quality of the assays, we can tailor the internal quality control (IQC) frequencies accordingly. An assay with high precision and accuracy, giving high sigma values requires fewer internal quality controls per run, more samples per run and fewer Westgard rules to adhere to. On the other hand, assays with higher imprecision or bias require more internal quality controls, smaller runs with fewer samples and more Westgard rules to follow. This is the concept advocated by Westgard in the newer Westgard Sigma Rules [14], [15] introduced. Thus this contributes to significant savings in terms of labor and cost for the well-established assays with a more focused approach for the troubled ones. Cost is a major constraint not only in the under developed regions of the world, but also for commercial laboratories elsewhere. The economy crunch which has started to engulf the various countries, especially so in this pandemic era, has made it apparent that organizations need to come up with cost-effective approaches in their running operations. Sigma metric is one such approach.

In this study, we have used three different methods to calculate sigma metrics of Alinity ci series in which bias has been calculated from the proficiency testing material data, method correlation and linearity experiment data generated during method validation studies. We have calculated sigma metrics of the assays performed on Alinity ci during initial validations, and this is part of an ongoing continuous exercise aimed at tailoring the use of internal quality controls.

## Materials and methods

This study was not related to either human or animal use and, therefore, was exempted from approval by the Institutional Review Board. It was conducted on 39 parameters across two Alinity c and two Alinity i analyzers during the months of June to September, 2018. Two sets of Alinity ci series on a3600 track were installed with pre and post analytics as part of total laboratory automation of the core clinical chemistry laboratory, and the data was generated during the method validation exercises performed on these analysers.

Alinity analytical platform was launched by Abbott Diagnostics in 2017. Alinity c is a totally automated clinical chemistry analyzer with indirect ion sensitive electrodes on integrated chip technology (ICT) for electrolytes, while Alinity i system is an immunoassay analyzer using paramagnetic microparticles and chemiluminescent detection based on Acridinium labeled conjugate. The clinical chemistry and electrolyte parameters included albumin, alkaline phosphatase (ALKP), alanine aminotransferase (ALT), amylase, aspartate aminotransferase (AST), blood urea nitrogen (BUN), calcium, chloride, cholesterol, CO_2_, creatinine phosphokinase (CPK), creatinine, direct bilirubin, gamma-glutamyl transferase (GGT), glucose, high density lipoprotein cholesterol (HDL-C) iron, lactate dehydrogenase (LDH), low density lipoprotein cholesterol (LDL-C), magnesium, phosphorus, potassium, sodium, total bilirubin, total protein, triglycerides, and uric acid, while the immunoassay parameters were alfa-fetoprotein (AFP) beta-human chorionic gonadotrophin (β-HCG), free tri-iodothyronine (FT_3_), free thyroxine (FT_4_), ferritin, follicle stimulating hormone (FSH), luteinizing hormone (LH), prolactin, thyroid stimulating hormone (TSH), total prostate specific antigen (PSA), and vitamins B12 and D. Sigma metrics of all analytes was calculated on both sets of analyzers, and the average was considered for the study purpose. Sigma metrics was calculated by the following formula:

***Σσ* = (TEα – Bias)/CV** (where *Σσ* is Sigma metric, TEα is the total allowable error and CV is the coefficient of variation).

- **Total allowable error:** All values for TEα were taken from Clinical and Laboratory Improvement Act (CLIA), College of American Pathologists (CAP) and National Cholesterol Education Program (NCEP), except for vitamin D for which TEα was taken from a reference study on biological variations (Table S1) [10]. TEα were kept similar or lower than the ones used in comparable studies [16], [17].
- **Precision:** Three levels of commercial quality controls were run in five replicates for five days on both Alinity ci instruments according to CLSI EP 15 guidelines [18] and coefficient of variation (CV%) was calculated.
- **Bias:** Bias was calculated by three different methods:

1.
**Proficiency testing**

Proficiency testing material of seven to ten levels obtained from College of American Pathologists were previously analyzed and stored at −40 °C. These were run on both Alinity c and i analyzers in three replicates. Results were compared to Abbott Architect peer group mean as Alinity was till the time of the study not included in CAP surveys. Bias was calculated as [(target mean − observed mean/target mean) × 100]. Cumulative bias was obtained by taking an average of all the biases.

2.
**Method correlation experiment**

Method correlation experiment was performed between Roche Cobas c311[(Roche Diagnostics International Ltd, Rotkreuz, Switzerland) for clinical chemistry], Nova 16[(Nova Biomedical Corporation 200 Prospect Street Waltham, Massachusetts, USA) for sodium, potassium, chloride, total CO_2_], Vitros Eci [Ortho Clinical Diagnostics, Rochester, NY) for β-HCG, ferritin, FSH, FT3, FT4, LH, prolactin, TSH and total PSA] and Elecsys e411 [(Roche Diagnostics International Ltd, Rotkreuz, Switzerland) for vitamin D, vitamin B12, AFP, intact parathyroid hormone and Alinity ci series. Forty samples were run on the existing method, and within 2 h of analysis were reanalyzed on both Alinity ci systems.

3.
**Linearity experiment**

Linearity studies were performed on all assays by running three replicates across seven levels using either commercial linearity material (clinical chemistry and electrolytes) or patient samples (immunoassay), except for analytes with multipoint calibrators. Bias was calculated from the difference between the expected and mean of observed values as given above. Bias and sigma metrics calculated from the linearity experiment are shown in Supplementary Table S2.

## Results

Concentration levels, precision, coefficient of variations and bias of these analytes are shown in Table 1. As can be seen, precision of all analytes are equal or less than 5.0%, while variability exists in the biases by different methods. Precision of clinical chemistry parameters and electrolytes is higher than immunoassays.

**Table 1:** CV and bias of analytes according to two methods.

Analytes	CV, %			BIAS, %	
	L1	L2	L3	Proficiency testing method	Alternate method comparison
**Clinical chemistry**					
ALB	1.062	0.835	0.599	3.49	1.41
ALKP	2.202	1.623	1.413	5.34	0.29
ALT	1.687	1.776	0.966	2.78	−0.53
AMY	2.289	0.913	0.694	3.59	−1.07
AST	1.628	1.046	0.876	5.52	−0.64
BUN	1.808	1.923	1.548	−1.77	1.16
Ca	1.221	0.961	0.967	−0.75	−0.27
CHOL	1.030	0.980	0.990	−0.47	−0.46
CO_2_	3.204	3.507	4.871	1.22	1.94
CPK	1.273	0.740	0.691	14.01	1.86
CREA	3.109	1.726	0.977	2.38	2.98
DB	1.512	2.490	1.172	−0.40	0.85
GGT	1.886	0.908	1.002	9.57	−1.20
GLU	0.975	0.538	0.335	1.15	−0.34
HDL-C	1.675	0.791	0.962	4.21	−1.65
IRON	1.052	0.627	0.910	3.18	−2.36
LDH	1.042	0.988	0.781	4.27	−0.50
LDL-C	0.950	0.643	1.435	1.31	−1.75
Mg	1.996	1.719	1.725	−2.85	−1.21
P	2.028	1.337	0.974	−0.69	−0.22
TB	0.916	2.195	2.174	0.63	−0.17
TG	2.145	0.943	0.853	−3.13	2.16
TP	0.725	0.772	0.689	0.47	−0.03
UA	2.124	0.922	0.814	−0.43	−0.42
**Immunoassay**					
AFP	3.351	3.123	3.230	−0.26	−1.62
βHCG	4.382	4.158	2.198	−4.35	0.77
FER	2.839	2.725	2.606	−5.65	−10.53
FSH	1.890	3.047	2.510	−0.05	−3.49
FT3	2.744	3.420	3.164	−6.64	−3.33
FT4	2.973	1.239	2.513	−5.83	1.79
LH	2.063	2.762	2.360	6.50	−4.63
Prolactin	2.241	2.667	2.667	3.64	3.11
Total PSA	1.370	1.910	2.274	−3.46	−7.30
TSH	3.157	4.085	2.767	6.14	−2.22
VIT B12	5.061	3.709	4.640	−2.93	−0.07
VIT D	4.833	4.952	3.337	−3.90	−6.40
**Electrolytes**					
Cl	0.647	0.706	0.583	1.51	1.42
K	1.856	1.173	0.842	0.43	0.41
Na	0.766	0.537	0.737	1.58	−0.07

ALB, albumin; ALKP, alkaline phosphatase; ALT, alanine aminotransferase; AMY, amylase; AST, aspartate aminotransferase; Ca, calcium; CHOL, cholesterol; CO_2_, carbon dioxide; CPK, creatinine phosphokinase; CREA, creatinine; DB, direct bilirubin; GGT, gamma glutamyl transferase; GLU, glucose; HDL, high density lipoprotein; LDH, lactate dehydrogenase; LDL, low density lipoprotein; Mg, magnesium; P, phosphorus; Total PSA, total prostatic specific antigen; TB, total bilirubin; TG, triglycerides; TP, total protein; UA, uric acid; AFP, alpha fetoprotein; βHCG, beta human chorionic gonadotrophin; FER, ferritin; FSH, follicle stimulating hormone; FT3, free triiodothyronine; FT4, free thyroxine; LH, luteinizing hormone; TSH, thyroid stimulating hormone; VIT B12, vitamin B12; VIT D, vitamin D; Cl, chloride K, potassium; Na, sodium.

Detailed sigma metric analysis is given in Table 2. In clinical chemistry, all parameters except BUN, demonstrated greater than six sigma values in one or more levels while BUN, CO_2_, creatinine, phosphorous and triglycerides gave values of less than Six Sigma in one or more levels and methods. Amongst immunoassays ferritin, prolactin and PSA demonstrated greater than six sigma values across all levels and methods, while other parameters demonstrated more than six sigma values at one or more levels across the different methods. No parameter in clinical chemistry or immunoassays was below three sigma level. Within electrolytes, potassium stood at greater than six sigma class across all levels and methods, while sodium was the only parameter to fall at below three sigma value at two levels in the proficiency testing method (Table 2).

**Table 2:** Sigma metric analysis of analytes via various methods on Alinity ci series.

Analytes	TEα	Source	Proficiency method			Alternate method comparison		
			L1	L2	L3	L1	L2	L3
**Clinical chemistry**								
ALB	10%	CLIA	6.13	7.79	10.86	8.09	10.29	14.33
ALKP	30%	CLIA	11.20	15.19	17.45	13.49	18.30	21.03
ALT	20%	CLIA	10.21	9.70	17.84	11.54	10.96	20.16
AMY	30%	CLIA	11.54	28.92	38.07	12.64	31.67	41.69
AST	20%	CLIA	8.89	13.84	16.52	11.89	18.50	22.10
BUN	9%	CLIA	4.00	3.76	4.67	4.34	4.08	5.06
Ca	9.72%	CLIA	7.34	9.33	9.28	7.74	9.83	9.78
CHOL	9%	NCEP	8.28	8.80	8.62	8.29	8.71	8.63
CO_2_	25%	CAP	7.42	6.78	4.88	7.20	6.57	4.73
CPK	30%	CLIA	12.56	21.60	23.12	22.11	38.01	40.70
CREA	15%	CLIA	4.06	7.32	12.93	3.87	6.97	12.31
DB	20%	CLIA	12.96	7.87	16.72	12.67	7.69	16.34
GGT	22.1%	RICOS	6.65	13.80	12.50	11.08	23.01	20.85
GLU	10%	CLIA	9.08	16.45	26.42	9.91	17.95	28.83
HDL-C	30%	CLIA	15.40	32.61	26.80	16.92	35.85	29.46
IRON	20%	CLIA	15.99	26.84	18.49	16.76	28.14	19.38
LDH	20%	CLIA	15.10	15.92	20.15	18.71	19.74	24.98
LDL-C	20%	CAP	19.69	29.09	13.02	19.22	28.40	12.72
Mg	25%	CLIA	11.09	12.88	12.84	11.92	13.84	13.79
P	10.7%	CAP	4.94	7.48	10.28	5.17	7.87	10.77
TB	20%	CLIA	21.14	8.83	8.91	21.63	9.03	9.12
TG	15%	NCEP	5.54	12.58	13.91	5.99	13.61	15.04
TP	10%	CLIA	13.15	12.35	13.83	13.76	12.92	14.46
UA	17%	CLIA	7.80	17.98	20.36	7.80	17.99	20.38
**Immunoassay**								
AFP	20%	RCPA	5.89	6.32	6.11	5.49	5.88	5.69
βHCG	30%	RiliBAK	5.85	6.17	11.67	6.67	7.03	13.30
FER	30%	CAP	8.57	8.94	9.34	6.86	7.15	7.47
FSH	20%	RCPA	10.55	6.55	7.95	8.73	5.42	6.58
FT3	17%	RICOS	3.78	3.03	3.27	4.98	4.00	4.32
FT4	16%	Spanish EQA minimum	3.42	8.21	4.05	4.78	11.47	5.66
LH	20%	RCPA	6.54	4.59	5.72	7.45	5.57	6.51
Prolactin	20%	RCPA	7.30	6.14	6.13	7.54	6.33	6.33
Total PSA	20%	Ricos desirable	12.07	8.66	7.27	11.98	8.59	7.22
TSH	23.7%	CLIA	8.70	6.72	9.92	6.81	5.26	7.76
VIT B12	30%	WSLH	5.35	7.30	5.83	5.91	8.07	6.45
VIT D	30%	Biological variation paper	5.40	5.27	7.82	4.88	4.76	7.07
**Electrolytes**								
Cl	5%	CLIA	5.39	4.94	5.98	5.53	5.06	6.13
K	17.97%	CLIA	9.45	14.96	20.84	9.46	14.97	20.86
Na	3.57%	CLIA	2.59	3.70	2.70	4.57	6.52	4.75

>6 
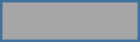
 ≥3–6 
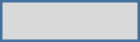
 <3 
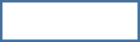
. ALB, albumin; ALKP, alkaline phosphatase; ALT, alanine aminotransferase; AMY, amylase; AST, aspartate aminotransferase; Ca, calcium; CHOL, cholesterol; CO_2_, carbon dioxide; CPK, creatinine phosphokinase; CREA, creatinine; DB, direct bilirubin; GGT, gamma glutamyl transferase; GLU, glucose; HDL, high density lipoprotein; LDH, lactate dehydrogenase; LDL, low density lipoprotein; Mg, magnesium; P, phosphorus; Total PSA, total prostatic specific antigen; TB, total bilirubin; TG, triglycerides; TP, total protein; UA, uric acid; AFP, alpha fetoprotein; βHCG, beta human chorionic gonadotrophin; FER, ferritin; FSH, follicle stimulating hormone; FT3, free triiodothyronine; FT4, free thyroxine; LH, luteinizing hormone; TSH, thyroid stimulating hormone; VIT B12, vitamin B12; VIT D, vitamin D; Cl, chloride; K, potassium; Na, sodium; CLIA 1992 was the source of total allowable errors for various analytes

**Figure 1:**
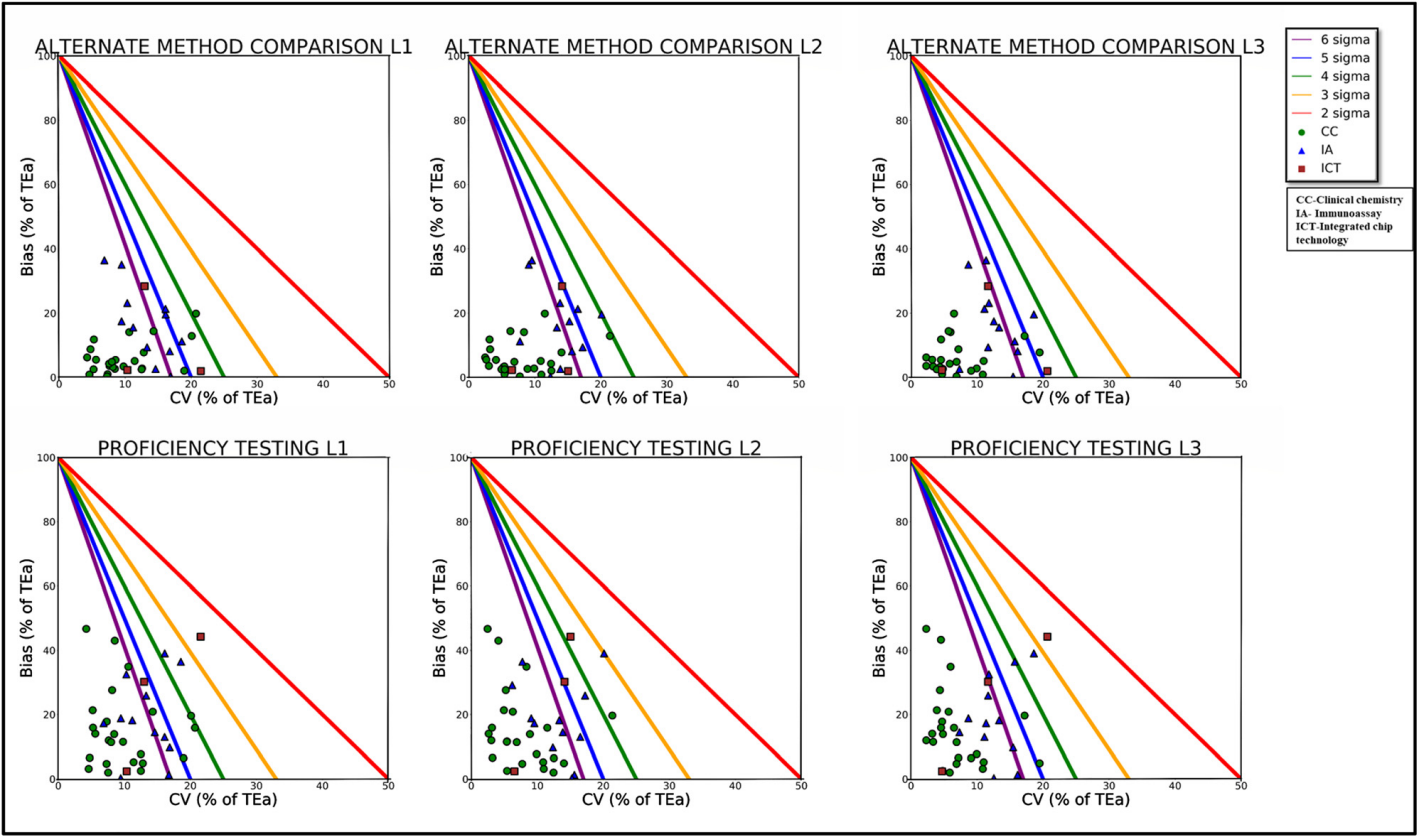
Normalized method decision chart displaying sigma metric values using two different methods.

Sigma metrics achieved in this study are comparable with other studies on Alinity as evident in Table 3. The Method Decision Charts (MEDx) made using Matplotlib package in Python3.6 at three concentration levels by the three different methods are displayed in Figure 1 and Figure S1.

**Table 3:** Comparison of studies on Alinity ci series.

Analytes	Westgard (2017)				Taher (2019)				Present study (2019)			
	TEα, %	Source	Conc.	*σ*	TEα, %	Source	Conc.	*σ*	TEα, %	Source	Conc.	*σ*
**Clinical chemistry**												
ALB	10	CLIA	2.91 g/dL	20.6	–	–		–	10	CLIA	2.99 g/dL	6.13
ALKP	30	CLIA	187.25 U/L	16.7	30	CLIA	151 U/L	10.6	30	CLIA	193.02 U/L	15.19
ALT	20	CLIA	30.2 U/L	10.8	20	CLIA	28 U/L	6	20	CLIA	26.08 U/L	10.21
AMY	30	CLIA	114.32 U/L	38.5	30	CLIA	132 U/L	27	30	CLIA	115.12 U/L	28.92
AST	20	CLIA	41.93 U/L	11.4	20	CLIA	40 U/L	8	20	CLIA	41.76 U/L	8.89
BUN	9	CLIA	38.82 mg/dL	4.24	9	CLIA	48.4 mg/dL	5.7	9	CLIA	43.22 mg/dL	3.76
Ca	9.72	CLIA	10.29 mg/dL	8.3	9.72	CLIA	9.82mg/dL	5.3	9.72	CLIA	9.89 mg/dL	9.33
CHOL	10	CLIA	154.99 mg/dL	9.06	–	–	–	–	9	NCEP	185.86 mg/dL	8.70
CO_2_	25	CAP	22.34 mEq/L	5.6	25	CAP	21 mEq/L	3.9	25	CAP	21.27 mEq/L	6.78
CPK	30	CLIA	148.21 U/L	26.2	–	–	–	–	30	CLIA	233.34 U/L	21.60
CREA	15	CLIA	2.00 mg/dL	6.9	–	–	–	–	15	CLIA	2.12 mg/dL	7.32
DB	44.5	Ricos desirable	0.4 mg/dL	11.2	–	–	–	–	20	CLIA	0.41 mg/dL	12.96
GGT	22.1	Ricos desirable	71.42 U/L	15.7	–	–	–	–	22.1	RICOS	59.94 U/L	13.80
GLU	10	CLIA	126.86 mg/dL	8.7	10	CLIA	118.8 mg/dL	5.7	10	CLIA	141.76 mg/dL	16.45
HDL	30	CLIA	51.77 mg/dL	13.6	–	–	–	–	30	CLIA	41.08 mg/dL	15.40
IRON	20	CLIA	103.03 μg/dL	14.4	–	–	–	–	20	CLIA	99.72 μg/dL	15.99
LDH	20	CLIA	127.98 U/L	5.5	–	–	–	–	20	CLIA	129.10 U/L	15.10
LDL	20	CAP	78.44 mg/dL	11.7	–	–	–	–	20	CAP	63.04 mg/dL	19.69
Mg	25	CLIA	2.22 mg/dL	17.5	25	CLIA	1.87 mg/dL	16.7	25	CLIA	2.32 mg/dL	12.88
P	10.7	CAP	4.24 mg/dL	5.8	10.7	CAP	–	5.4	10.7	CAP	4.37 mg/dL	7.48
TB	20	CLIA	2.78 mg/dL	9.14	20	CLIA	2.81 mg/dL	7.6	20	CLIA	2.57 mg/dL	8.83
TG	25	CLIA	150.79 mg/dL	27.6	–	–	–	–	15	NCEP	142.2 mg/dL	12.58
TP	10	CLIA	5.09 g/dL	7.65	10	CLIA	4.9 g/dL	6.2	10	CLIA	4.48 g/dL	13.15
UA	17	CLIA	2.49 mg/dL	15.6	–	–	–	–	17	CLIA	2.34 mg/dL	7.80
**Immunoassay**												
AFP	–	–		–	–	–	–	–	20	RCPA	5.42 IU/L	5.89
βHCG	30	RiliBAK	24.45 IU/mL	5.48	30	RiliBAK	26 IU/L	4.4	30	RiliBAK	4.49 IU/L	5.85
FER	–	–	–	–	–	–	–	–	30	CAP	32.83 ng/mL	8.57
FSH	–	–	–	–	–	–	–	–	20	RCPA	35.73 U/L	7.95
FT3	17	Ricos minimum	6.15 pg/mL	3.12	–	–	–	–	17	RICOS	5.08 pg/mL	3.78
FT4	16	Spanish EQA minimum	1.16 ng/dL	5.94	–	–	–	–	16	Spanish EQA minimum	11.34 ng/dL	3.42
LH	–	–		–	–	–	–	–	20	RCPA	38.43 U/L	5.72
Prolactin	29.4	Ricos desirable	38.75 ng/mL	8.83	–	–	–	–	20	RCPA	41.44 ng/mL	6.13
TSH	23.7	Ricos desirable	0.31 μIU/mL	10.9	23.7	Ricos desirable	0.16 µIU/mL	10	23.70	Ricos desirable	0.051 μIU/mL	5.56
PSA^a^	33.6	Ricos desirable	0.39 ng/mL	5.88	–	–	–	–	20	CLIA	3.40 ng/mL	8.66
VIT B12	–	–		–	–	–	–	–	30	WSLH	294.68 pg/mL	5.35
VIT D	30	Biological variation paper	21.14 ng/mL	7.17	–	–	–	–	30	Biological variation paper	23.28 ng/mL	5.27
**Electrolytes**												
Cl	5	CLIA	94.65 mmol/L	6.57	5	CLIA	96 mmol/L	5.2	5	CLIA	96.73 mmol/L	4.94
K	17.97	CLIA	2.78 mmol/L	11.9	18	CLIA	2.7 mmol/L	11.8	17.97	CLIA	2.64 mmol/L	9.45
Na	3.57	CLIA	112.03 mmol/L	4.2	4	CLIA	121 mmol/L	4.1	3.57	CLIA	121.95 mmol/L	2.59

ALB, albumin; ALKP, alkaline phosphatase; ALT, alanine aminotransferase; AMY, amylase; AST, aspartate aminotransferase; Ca, calcium; CHOL, cholesterol; CO_2_, carbon dioxide; CPK, creatinine phosphokinase; CREA, creatinine; DB, direct bilirubin; GGT, gamma glutamyl transferase; GLU, glucose; HDL, high density lipoprotein; LDH, lactate dehydrogenase; LDL, low density lipoprotein; Mg, magnesium; P, phosphorus; Total PSA, total prostatic specific antigen; TB, total bilirubin; TG, triglycerides; TP, total protein; UA, uric acid; AFP, alpha fetoprotein; βHCG, beta human chorionic gonadotrophin; FER, ferritin; FSH, follicle stimulating hormone; FT3, free triiodothyronine; FT4, free thyroxine; LH, luteinizing hormone; TSH, thyroid stimulating hormone; VIT B12, vitamin B12; VIT D, vitamin D; Cl, chloride; K, potassium; Na, sodium; CLIA 1992 was the source of Total allowable errors for various analytes. ^a^Westgard had analyzed free PSA while in the present study total PSA has been analyzed resulting in different concentrations. Thus a comparison is unlikely.

## Discussion

Sigma metrics analysis has emerged as an easy to use and objective tool for monitoring and “quantitating” the quality of test assays. It can be calculated easily from routine quality control values generated in the laboratories at no additional expense. Moreover, it can be implemented as a tool to tailor internal quality control policy and to reduce the daily frequency and number of controls, serving as a cost reducing activity. In the current financial crisis gripping the world where laboratories are struggling to balance the quality monitoring measures with the expenses entailed, application of sigma metric based QC rules finds a new dimension in their utility.

Our initial validation studies on Alinity ci (Abbott Diagnostics, Abbott Gmbh & Co. KG, Wiesbaden, Germany) was very promising and demonstrated high sigma metrics for majority of the assays. All three methods performed well, but the results were lower with the proficiency testing method. This could be because the PT samples were stored at −40 °C before use. To overcome this challenge, sample integrity was first confirmed by running the samples on the existent analyzers and comparing the results to the participant summary results. They were then repeated on Alinity ci within 2 h. Although we used proficiency testing, alternate method comparison and linearity studies to generate sigma metrics, we recommend proficiency testing as a more standardized tool to compare one’s method with other laboratories around the globe on a continuous basis. Alternate method comparison is useful during the initial assessment phase only and at times difficult to adopt in immunoassays, while linearity studies are more useful for measuring range and calibration verification studies than for bias estimation.

A major determinant of the sigma metric calculation is the choice of TEα [19]. The more expansive the limit, the better sigma would be achieved, giving a false sense of security and over estimation of the assay quality. The use of stringent limits reduces the sigma levels and gives a better and truer picture of the assay quality. We have tried to use similar to or lower TEαs than the comparative studies on Alinty ci. Westgard [17] and Tahir et al. [16] have defined a sequence whereby they have used lowest realistically possible error limits of the assays. We have kept the same limits in order to compare sigma scale achieved by us with the initial analyses done within factory settings or in the field by company researchers. In certain assays such as direct bilirubin (20 vs. 44.5%), triglycerides (15 vs. 25%) and prolactin (20 vs 29.4%), we have chosen lower allowable error limits than the ones selected in the reference studies, yet achieved six sigma levels. The total allowable goals put forward by CLIA in 2019 are a step to raise the bar even more and we intend to use these in our further assessments.

Another noteworthy element in the current study was its scope. We included multiple analytes which were not part of the earlier studies by Westgard et al. [17] (AFP, ferritin, FSH, LH and vitamin B12) and Taher et al. [16] (albumin, cholesterol, CPK, creatinine, direct bilirubin, GGT, HDL, iron, LDH, LDL, triglycerides, uric acid, AFP, ferritin, FSH, FT3, FT4, LH, prolactin, PSA, vitamins B12 and D).

Clinical chemistry analytes generally demonstrated better sigma levels than immune assay parameters. This was because of the higher precision inherent in these assays compared to immunoassays. The variability in the sigma levels across different methods was due to the variation in the biases generated, as the precision used in the calculations was constant, being derived from the internal quality controls data.

There were few limitations to the study. Firstly, the proficiency testing results were compared to Architect peer group due to the fact that Alinity CAP participation results were not existent at the time of the study. Secondly, the study was performed over a short span of time with EP 15 for generating the precision data and by using a single lot of reagents, calibrators and controls. The experience now after going live with these assays is a different story with real time peer group analysis in CAP surveys, and varied lots of reagents, calibrators, controls, environmental conditions and operators.

## Conclusions

Alinity ci series generated acceptable sigma metrics for all routine clinical chemistry and immunoassay parameters. Sigma metric calculation is a practical tool for assessing the quality of an analyte based on its precision and accuracy which can help the laboratories to modify and taper the IQC frequencies. It enables laboratories to work in a more cost effective manner without compromising on patient safety. The present study was conducted using different total allowable errors limits. However, with the introduction of the revised and restricted 2019 CLIA total allowable error goals, we propose that laboratories should universally adopt these to calculate their sigma metrics.

## Supplementary Material

Supplementary Material Details
