# Supplementary material for: Sigma metrics of Alinity ci system – a study on thirty-nine clinical chemistry and immunoassay parameters
Source: Adv Lab Med. 2021 Apr 1;2(2):267–75. doi: 10.1515/almed-2021-0001 (PMC10197361; doi:10.1515/almed-2021-0001)

**SUPPLEMENTARY FILE**

**Table S1: Various sources of Total Allowable Errors (TEαs)**

| ANALYTE | CLIA | | CAP | RCPA | RICOS | | RiliBÄK | SPANISH EQA MINIMUM | NCEP | BIOLOGICAL VARIATION PAPER |
| --- | --- | --- | --- | --- | --- | --- | --- | --- | --- | --- |
|  | **2019** | **1992** |  |  | **DESIRABLE** | **OPTIMAL** |  |  |  |  |
| CLINICAL CHEMISTRY | | | | | | | | | | |
| ALB | 8% | 10% | 10% | 6% | 4.07% | 23.3% | - | 14% | - | - |
| ALKP | 20% | 30% | 30% | 12% | 12.04% | - | 18% | 31% | - | - |
| ALT | 15% | 20% | 20% | 12% | 27.48% | 13.7% | 21% | 23% | - | - |
| AMY | 10% | 30% | 30% | 10% | 14.6% | 7.3% | - | 35% | - | - |
| AST | - | 20% | 20% | 12% | 16.69% | 8.3% | - | 21% | - | - |
| BUN | 9% | 9% | 9% | 12% | 15.5% | 7.8% | 20% | 19% | - | - |
| Ca | 9.72% | 9.72% | 1mg/dL. | 4% | 2.4% | - | 10% | 11% | - | - |
| CHOL | 10% | 10% | 10% | 6% | 9.01% | 4.5% | 13% | 11% | 9% | - |
| CO_2_ | 20% | - | 25% | 8% | 5.7% | - | 12% | - | - | - |
| CPK | 20% | 30% | 30% | 12% | 30.3% | 15.2% | 20% | 24% | - | - |
| CREA | 10% | 15% | 15% | 8% | 8.87% | 7.7% | 20% | 20% | - | - |
| DB | - | 20% | 20% | 20% | 44.5% | - | - | - | - | - |
| GGT | 15% | 20% | 3SD | 12% | 22.1% | 11.1% | 21% | 22% | - | - |
| GLU | 8% | 10% | 10% | 8% | 6.96% | - | 15% | 11% | - | - |
| HDL-C | 20% | 30% | 30% | 12% | 11.63% | - | - | 33% | 13% | - |
| IRON | 15% | 20% | 20% | 12% | 30.7% | 15.3% | - | 24% | - | - |
| LDH | 15% | 20% | 20% | 8% | 11.4% | 5.7% | 18% | 26% | - | - |
| LDL-C | 20% | - | 20% | 10% | 11.9% | - | - | - | 12% | - |
| Mg | 15% | 25% | 25% | 8% | 4.8% | - | 15% | - | - | - |
| P | 10% | - | 10.7% | 8% | 10.11% | 5.1% | 16% | 17% | - | - |
| TB | 20% | 20% | 20% | 12% | 26.94% | 13.5% | 22% | 24% | - | - |
| TG | 15% | 25% | 25% | 12% | 28% | 13% | 16% | 18% | 15% | - |
| TP | 8% | 10% | 10% | 5% | 3.63% | - | 10% | 12% | - | - |
| UA | 10% | 17% | 17% | 8% | 11.9% | 6% | 13% | 17% | - | - |
| IMMUNOASSAY | | | | | | | | | | |
| AFP | 20% | 3SD | 3SD | 20% | 21.9% | 10.9% | - | - | - | - |
| βHCG | 18% | 3SD | 3SD | 15% | - | - | 30% | - | - | - |
| FER | 20% | - | 30% | 15% | - | 8.4% | 25% | 21% | - | - |
| FSH | 18% | - | - | 20% | 21.19% | 10.6% | 21% | 14% | - | - |
| FT3 | 30% | 3SD | 3SD | 20% | 11.3% | 17% | 20% | 24% | - | - |
| FT4 | 15% | 3SD | 20% | 12% | 8.0% | - | 20% | 16% | - | - |
| LH | 20% | - | - | 20% | 27.92% | 14% | - | 17% | - | - |
| PROLACTIN | 20% | - | - | 20% | 29.4% | 14.7% | - | 22% | - | - |
| TOTAL PSA | 20% | - | - | 8% | 33.6% | 16.8% | 25% | 17% | - | - |
| TSH | 20% | 3SD | 3SD | 20% | 23.7% | 11.9% | - | 15% | - | - |
| VIT B12 | 25% | 30% | 3SD | 15% | - | - | - | - | - | - |
| VIT D | - | - | - | 15% | - | - | - | - | - | 30% |
| ELECTROLYTES | | | | | | | | | | |
| Cl | 5% | 5% | 5% | 3% | 1.5% | - |  | 9% | - | - |
| K | 0.3mmol/L | 17.97% | - | 5% | 5.61% | - |  | 8% | - | - |
| Na | 3.57% | 3.57% | - | 2% | 0.73% | - |  | 5% | - | - |

ALB- Albumin; ALKP- Alkaline Phosphatase; ALT- Alanine Aminotransferase; AMY- Amylase; AST- Aspartate aminotransferase; Ca- Calcium; CHOL- Cholesterol; CO_2_- Carbon dioxide; CPK-Creatinine Phosphokinase; CREA- Creatinine; DB- Direct Bilirubin; GGT-Gamma Glutamyl Transferase; GLU- Glucose; HDL-High Density Lipoprotein; LDH-Lactate Dehydrogenase; LDL-Low Density Lipoprotein; Mg-Magnesium; P-Phosphorus; TOTAL PSA- Total Prostatic Specific Antigen; TB- Total Bilirubin; TG-Triglycerides; TP- Total protein; UA-Uric acid; AFP-Alpha fetoprotein; βHCG- Beta Human Chorionic Gonadotrophin; FER-Ferritin; FSH-Follicle Stimulating Hormone; FT3-Free Triiodothyronine; FT4-Free Thyroxine; LH-Luteinizing Hormone; TSH-Thyroid Stimulating Hormone; VIT B12-Vitamin B12; VIT D- Vitamin D; Cl-Chloride K-Potassium; Na-Sodium

The grey highlighted cells mark the TEα that was considered in the present study.

**Table S2: Bias and Sigma metric of the analytes according to linearity method**

| **ANALYTES** | **BIAS** | **TEα** | **SOURCE** | **SIGMA METRIC** | | |
| --- | --- | --- | --- | --- | --- | --- |
|  |  |  |  | **L1** | **L2** | **L3** |
| **ALB** | -0.63 | 10% | CLIA | 8.83 | 11.23 | 15.64 |
| **ALKP** | -0.18 | 30% | CLIA | 13.54 | 18.37 | 21.10 |
| **ALT** | 0.06 | 20% | CLIA | 11.82 | 11.22 | 20.65 |
| **AMY** | -0.06 | 30% | CLIA | 13.08 | 32.78 | 43.15 |
| **AST** | -0.02 | 20% | CLIA | 12.27 | 19.10 | 22.81 |
| **BUN** | -0.14 | 9% | CLIA | 4.90 | 4.61 | 5.72 |
| **Ca** | -0.42 | 9.72% | CLIA | 7.62 | 9.68 | 9.62 |
| **CHOL** | 0.20 | 9% | NCEP | 8.55 | 8.98 | 8.89 |
| **CO2** | 0.47 | 25% | CAP | 7.66 | 7.00 | 5.04 |
| **CPK** | 0.66 | 30% | CLIA | 23.05 | 39.63 | 42.43 |
| **CREA** | 0.00 | 15% | CLIA | 4.82 | 8.69 | 15.36 |
| **DB** | 0.49 | 20% | CLIA | 12.91 | 7.84 | 16.65 |
| **GGT** | 1.30 | 22.1% | RICOS | 11.03 | 22.90 | 20.75 |
| **GLU** | 0.15 | 10% | CLIA | 10.10 | 18.29 | 29.38 |
| **HDL-C** | -0.57 | 30% | CLIA | 17.57 | 37.21 | 30.58 |
| **IRON** | 0.02 | 20% | CLIA | 18.99 | 31.88 | 21.96 |
| **LDH** | -0.06 | 20% | CLIA | 19.13 | 20.17 | 25.53 |
| **LDL-C** | -0.33 | 20% | CAP | 20.72 | 30.62 | 13.71 |
| **Mg** | -1.77 | 25% | CLIA | 1164 | 13.51 | 13.46 |
| **P** | -0.08 | 10.7% | CAP | 5.24 | 7.94 | 10.91 |
| **TB** | -0.72 | 20% | CLIA | 21.04 | 8.79 | 8.87 |
| **TG** | -0.10 | 15% | NCEP | 6.95 | 15.79 | 17.45 |
| **TP** | 0.28 | 10% | CLIA | 13.41 | 12.59 | 14.10 |
| **UA** | 0.53 | 17% | CLIA | 7.75 | 17.87 | 20.25 |
| **IMMUNOASSAY** | | | | | | |
| **AFP** | 0.00* | 20% | RCPA | 5.97 | 6.40 | 6.19 |
| **βHCG** | 0.00* | 30% | RiliBAK | 6.85 | 7.22 | 13.65 |
| **FER** | 1.20 | 30% | CAP | 10.14 | 10.57 | 11.05 |
| **FSH** | 2.05 | 20% | RCPA | 9.50 | 5.89 | 7.15 |
| **FT3** | 0.00* | 17% | RICOS | 6.20 | 4.97 | 5.37 |
| **FT4** | 0.00* | 16% | Spanish EQA Minimum | 5.38 | 12.92 | 6.37 |
| **LH** | 0.00* | 20% | RCPA | 9.69 | 7.24 | 8.48 |
| **PROLACTIN** | 0.34 | 20% | RCPA | 8.77 | 7.37 | 7.37 |
| **TOTAL PSA** | 0.63 | 20% | Ricos Desirable | 14.14 | 10.14 | 8.52 |
| **TSH** | 2.02 | 23.7% | CLIA | 6.87 | 5.31 | 7.84 |
| **VIT B12** | 0.00* | 30% | WSLH | 5.93 | 8.09 | 6.47 |
| **VIT D** | 0.00* | 30% | Biological Variation Paper | 6.21 | 6.06 | 8.99 |
| **ELECTROLYTES** | | | | | | |
| **Cl** | 0.00 | 5% | CLIA | 7.72 | 7.08 | 8.57 |
| **K** | 0.02 | 17.97% | CLIA | 9.67 | 15.30 | 21.33 |
| **Na** | -0.43 | 3.57% | CLIA | 4.10 | 5.85 | 4.27 |

**>6 ≥3-6 <3**

ALB- Albumin; ALKP- Alkaline Phosphatase; ALT- Alanine Aminotransferase; AMY- Amylase; AST- Aspartate aminotransferase; Ca- Calcium; CHOL- Cholesterol; CO_2_- Carbon dioxide; CPK-Creatinine Phosphokinase; CREA- Creatinine; DB- Direct Bilirubin; GGT-Gamma Glutamyl Transferase; GLU- Glucose; HDL-High Density Lipoprotein; LDH-Lactate Dehydrogenase; LDL-Low Density Lipoprotein; Mg-Magnesium; P-Phosphorus; TOTAL PSA- Total Prostatic Specific Antigen; TB- Total Bilirubin; TG-Triglycerides; TP- Total protein; UA-Uric acid; AFP-Alpha fetoprotein; βHCG- Beta Human Chorionic Gonadotrophin; FER-Ferritin; FSH-Follicle Stimulating Hormone; FT3-Free Triiodothyronine; FT4-Free Thyroxine; LH- Luteinizing Hormone; TSH-Thyroid Stimulating Hormone; VIT B12-Vitamin B12; VIT D- Vitamin D; Cl-Chloride K-Potassium; Na-Sodium; CLIA 1992 was the source of Total allowable errors for various analytes *Analytes with multipoint calibrators

**Figure S1: Normalized Method Decision Chart displaying Sigma metric values using linearity method**


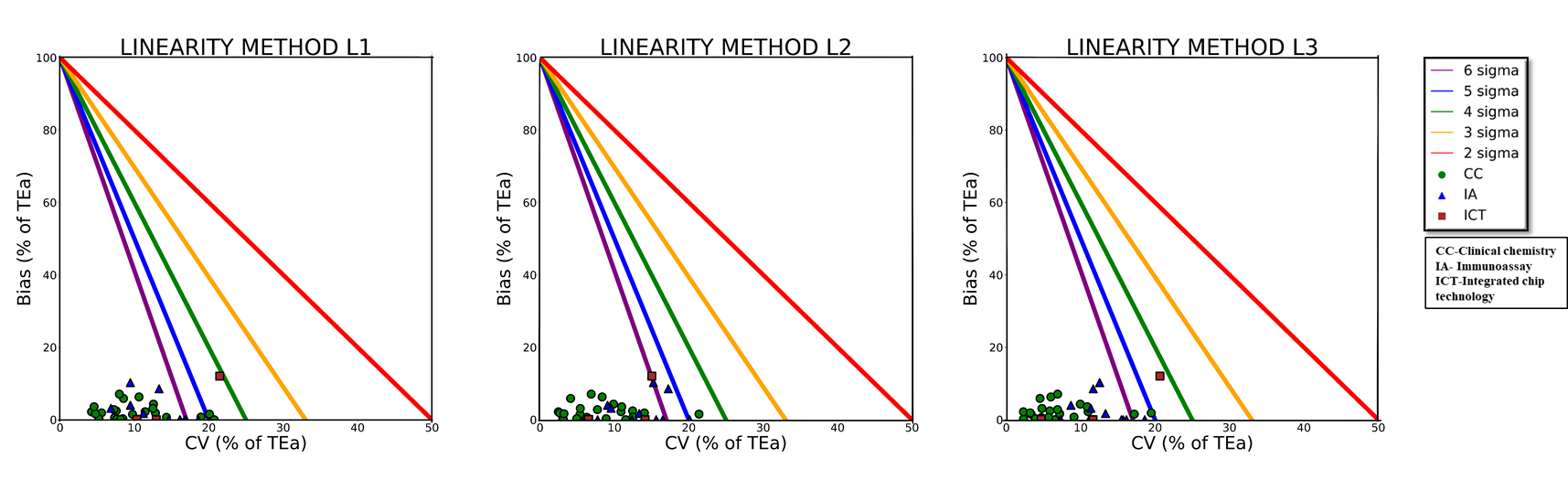

Supplement: Supplementary file 1 — Supplementary Material Details [file j_almed-2021-0001_suppl_001.docx]
